# Prognostic impact of *IKZF1* deletion in adults with common B-cell acute lymphoblastic leukemia

**DOI:** 10.1186/s12885-016-2300-7

**Published:** 2016-04-11

**Authors:** Qiu-Mei Yao, Kai-Yan Liu, Robert Peter Gale, Bin Jiang, Yan-Rong Liu, Qian Jiang, Hao Jiang, Xiao-Hui Zhang, Mei-Jie Zhang, Shan-Shan Chen, Xiao-Jun Huang, Lan-Ping Xu, Guo-Rui Ruan

**Affiliations:** Beijing Key Laboratory of Hematopoietic Stem Cell Transplantation, Peking University People’s Hospital and Institute of Hematology, 11 Xi-Zhi-Men South Street, 100044 Beijing, China; Haematology Research Center, Division of Experimental Medicine, Department of Medicine, Imperial College London, London, UK; Division of Biostatistics, Medical College of Wisconsin, Milwaukee, USA; Peking-Tsinghua Center for Life Sciences, Beijing, China

**Keywords:** *IKZF1*, Acute lymphoblastic leukemia, *BCR-ABL1*, Chemotherapy, Allotransplant

## Abstract

**Background:**

Interrogate the impact of *IKZF1* deletion on therapy-outcomes of adults with common B-cell acute lymphoblastic leukemia.

**Methods:**

One hundred sixty-five consecutive adults with common B-cell ALL were tested for *IKZF1* deletion and for BCR/ABL. Deletions in *IKZF1* were detected using multiplex RQ-PCR, multiplex fluorescent PCR, sequence analysis and multiplex ligation-dependent probe amplification (MLPA). *BCR/ABL* was detected using RQ-PCR. All subjects received chemotherapy and some also received an allotransplant and tyrosine kinase-inhibitors. Multivariate analyses were done to identify associations between *IKZF1* deletion and other variables on non-relapse mortality (NRM), cumulative incidence of relapse (CIR), leukemia-free survival (LFS) and survival.

**Results:**

Amongst subjects achieving complete remission those with *IKZF1* deletion had similar 5-year non-relapse mortality (NRM) (11 % [2–20 %] vs. 16 % [4–28 %]; *P* = 0.736), a higher 5-year cumulative incidence of relapse (CIR) (55 % [35–76 %] vs. 25 % [12–38 %]; *P* = 0.004), and worse 5-year leukemia-free survival (LFS) (33 % [16–52 %] vs. 59 % [42–73 %]; *P* = 0.012) and survival (48 % [33–62 %] vs. 75 % [57–86 %]; *P* = 0.002). In multivariate analyses *IKZF1* deletion was associated with an increased relapse (relative risk [RR] =2.7, [1.4–5.2]; *P* = 0.002), a higher risk of treatment-failure (inverse of LFS; *RR* = 2.1, [1.2–3.6]; *P* = 0.007) and a higher risk of death (*RR* = 2.8, [1.5–5.5]; *P* = 0.002). The adverse impact of *IKZF1* deletion on outcomes was stronger in subjects without vs. with *BCR-ABL1* and in subjects receiving chemotherapy-only vs. an allotransplant.

**Conclusions:**

*IKZF1* deletion was independently-associated with a higher relapse risk and worse LFS and survival in adults with common B-cell ALL after adjusting for other prognostic variables and differences in therapies. These data suggest *IKZF1* deletion may be a useful prognostic variable in adults with common B-cell ALL, especially in persons without *BCR-ABL1* and those receiving chemotherapy-only. Transplants appear to overcome the adverse impact of *IKZF1* deletion on therapy-outcomes but confirmation in a randomized study is needed. The trial was registered in 2007 with the Beijing Municipal Government (Beijing Municipal Health Bureau Registration N: 2007–1007).

## Background

About 85 % of cases of adult acute lymphoblastic leukemia (ALL) develop from B-cells. Identification of biological determinants of treatment-outcomes is incomplete. Consensus prognostic variables include age, gender, WBC level at diagnosis, cytogenetic abnormalities, *BCR-ABL1*, central nervous system (CNS) leukemia, time to initial response and detection of measureable residual disease (MRD) after induction and consolidation therapies [1–5]. However, analyses of receiver-operator characteristic (ROC) curves indicate only about one-half of the variability in subject-level outcomes is explained by known prognostic variables [6].

Genome-wide analyses indicate *IKZF1* mutations are common in ALL and are associated with *BCR-ABL1* [7, 8]. *IKZF1* encodes the Ikaros transcription factor, a member of a family of zinc-finger nuclear proteins required for normal lymphoid development [9]. Intra-genic deletions in *IKZF1* generate aberrant isoforms [8, 10]. The role of *IKZF1* deletions is extensively-evaluated in children with ALL and is the most common genetic marker associated with a poor prognosis [11–15]. *IKZF1* mutations are also common in adults with B-cell ALL and are also associated with poor prognosis in persons with concurrent *BCR-ABL1* [16–18]. Few data are available on the role of *IKZF1* deletions in adults with common B-cell ALL. In preliminary analyses we found frequent *IKZF1* deletions in this population. Based on these data we interrogated associations between *IKZF1* deletion and prognosis in a series of adults with common B-cell ALL.

## Methods

### Subjects

Between April, 2007 and December, 2012 165 newly-diagnosed subjects ≥14 years old with common B-cell ALL were seen at Peking University People’s Hospital, Peking University Institute of Hematology (Beijing, China). 3 subjects with severe co-morbidities were ineligible for induction chemotherapy (2 had an *IKZK1* deletion) and excluded from further analyses. The 162 eligible subjects received remission-induction chemotherapy. Those achieving complete remission received 2 cycles of consolidation chemotherapy after which they could continue receiving chemotherapy or receive an allotransplant based on donor availability, finances and physician and subject choice. Physicians and subjects were blinded to results of *IKZF1*-testing. Subjects were followed until death, loss to follow-up or December, 2014. Written informed consent was obtained from adult subjects and from parents of minors in accordance with the Declaration of Helsinki. The study was approved by the Ethics Committee of Peking University People’s Hospital. Subjects receiving post-remission chemotherapy were included in a Chinese Clinical Trial registered in 2007 with the Beijing Municipal Government (Beijing Municipal Health Bureau Registration N: 2007–1007).

### Diagnosis and response criteria

Diagnosis of ALL was based on ≥20 % bone marrow lymphoblasts. Common-B-cell type was defined as cases with CD10, CD19, CD22 and CD79a expression and no surface immunoglobulin by flow cytometry [19]. Complete remission was defined bone marrow lymphoblasts < 5 %, neutrophils >1.0 × 10E + 9/L, platelets >100 × 10E + 9/L, no extra-medullary disease and no loss of these features for >4 weeks. Relapse was defined as leukemia recurrence at any site in persons in complete remission.

### Cytogenetic and molecular analyses

Cytogenetic analyses were performed by using standard G-banding [20]. *BCR-ABL1* transcripts, *MLL* rearrangement and *E2A/PBX1* transcripts were detected with quantitative real-time polymerase chain reaction (RQ-PCR) [21, 22]. *IKZF1* deletions were detected using multiplex RQ-PCR, multiplex fluorescent PCR, sequence analysis [23] and multiplex ligation-dependent probe amplification (MLPA) (MRC-Holland, Amsterdam, Netherlands) performed using SALSA MLPA kit P202-B1 *IKZF1*. Focal *IKZF1* gene deletions (deletions of exons 4–7, 4–8, 2–7, 2–8 and other focal deletions identified by MLPA analysis) were defined as *IKZF1* deletion in the present study.

### Chemotherapy

One hundred six subjects without BCR/ABL received induction chemotherapy with 1–2 cycles of CODPL (cyclophosphamide, vincristine, daunorubicin, prednisone and L-asparaginase). 56 subjects with *BCR-ABL1* received induction chemotherapy with the same regimen without L-asparaginase and 48 also received imatinib, 400 mg/d, beginning at diagnosis. 5 were later switched to dasatinib, 1 to nilotinib and 1 to ponatinib because of resistant mutations. Data regarding *IKZF1* deletion were not used to determine induction chemotherapy. Subjects achieving complete remission (CR) received 2 cycles consolidation chemotherapy with hyper-CVAD (cyclophosphamide, vincristine, doxorubincin, dexamethasone, cytarabine and methotrexate). All subjects received CNS prophylaxis with intrathecal methotrexate, cytarabine and dexamethasone for ≥8 doses during induction and consolidation therapy. Details of these regimens are reported [24]. Subjects remaining in CR continued receiving chemotherapy until the completed 6 more cycles, received an allotransplant or relapsed. Post-consolidation therapy was with 6-mercaptopurine, 50 mg/mE2/d PO and methotrexate, 20 mg/mE2/d PO, each once weekly. 28 subjects with *BCR-ABL1* receiving an allotransplant also received posttransplant tyrosine kinase-inhibitor (TKI) therapy. 17 subjects with *BCR-ABL1* receiving post-consolidation chemotherapy but no allotransplant also received post-consolidation TKI therapy. Decisions to give or not to give TKI therapy post-consolidation or posttransplant were based on finances and subject and/or physician preference and without knowing results of *IKZF1* deletion-testing.

### Allotransplants

Ninety-two subjects (64 %) received an allotransplant, 24 (26 %) from an HLA-identical sibling and 68 (74 %) from a HLA-haplotype-mismatched related donor (1 HLA-antigen mismatch [*N* = 4], 2 HLA-antigen mismatches [*N* = 20] and 3 HLA-antigen mismatches [*N* = 44]). Conditioning was with busulfan and cyclophosphamide. Anti-thymocyte globulin was given pretransplant when the donor was a HLA-haplotype-mismatched relative. Donors received recombinant human G-CSF after which bone marrow and blood cells were collected and infused into the recipient. Graft-vs.-host disease prophylaxis was with cyclosporine, mycophenolatemofetil and short-term methotrexate. Haematological relapses were treated with donor mononuclear cells infusions. Some subjects received a 2^nd^ transplant from the same or a different donor. Details are reported [25, 26].

### Endpoints and statistical analyses

Haematologic response was analyzed weekly for the first 3 months and monthly thereafter. Cytogenetic and molecular responses were analyzed every 3 months for the first 6 months and every 6–12 months thereafter. Leukemia-free survival (LFS) was calculated from the date of 1^st^ complete remission to the date of first relapse or death in complete remission. Survival was calculated from 1^st^ complete remission to the date of death from any cause. Observations were censored at the date of last contact or December, 2014 when no events were observed. In survival analyses subjects receiving an allotransplant who relapsed were censored at the time they received a donor blood cell infusion or a second allotransplant. Last follow-up was December, 2014. Relapse was defined as 1^st^ hematologic relapse regardless of site. Independence of categorized parameters was calculated using Chi-square test (or Fisher Exact test). Distribution of continuous variables was calculated using Wilcoxon two sample tests. Survival functions were estimated by the Kaplan-Meier method and compared by the log-rank test. Cumulative incidences were estimated for NRM and relapse to accommodate competing risks. NRM was a competing risk for relapse and death from any cause was a competing risk for relapse. A Cox proportional hazard regression model was used to determine associations between *IKZF1* deletion and other variables with relapse, NRM, treatment-failure and death. The variables to be considered in the multivariate models were: *IKZF1* deletion (Y/N), *BCR-ABL1* (Y/N), gender, age (≥ vs. < 35 years), WBC (≥ vs. < 30 × 10E + 9/L), hypo-diploid karyotype (Y/N), complex karyotype and treatment (chemotherapy vs. allotransplant). CNS, *MLL* rearrangement or *E2A-PBX1* transcripts and TKI were not considered in the multivariate analysis because of either too few subjects in a category or missing data. Assumption of proportional hazards for each factor in the Cox model was tested using time-dependent covariates. A stepwise model selection approach was used to identify all significant risk factors. Each step of model building contained the main effect for *IKZF1* deletion. Factors which are significant at a *P* = 0.05 level were kept in the final model. Potential interactions between main effect and all significant risk factors were tested. Analyses were performed by SPSS software version 19.0 (Chicago, IL, USA), Graphpad Prism 5.01 (San Diego, California, USA) and R version 3.1.2. Difference with *P* < 0.05 was considered significant.

## Results

### Subjects

Sixty-eight *IKZF1* local deletions were detected of subjects achieving complete remission including 32 (47 %) subjects with type Δ4–7, 17 (25 %) with type Δ2–7, 3 with type Δ4–8, 10 (15 %) with type Δ2-8 and 6 with other deletions. 6 other deletions identified by MLPA analysis were undetectable by multiplex PCR. Proportions of dominant negative isoforms (Δ4–7 deletion) and haplotype-insufficiency (other local deletion types) were comparable. The proportion of Δ4–7 deletion in subjects with *BCR-ABL1* was higher than in subjects without *BCR-ABL1* (20/31 vs. 12/37; *P* = 0.008). Baseline subject- and disease-related variables of subjects achieving CR are shown in Table 1. Subjects with *IKZF1* deletion were older (34 years [15–70] vs. 22 [14–59]; *P* = 0.045), had a significantly higher WBC (14 [2–435] vs. 9 [0.5–168]; *P* = 0.008) and were more likely to have *BCR-ABL1* (75 % vs. 25 %; *P* < 0.01) and t (9; 22) (72 % vs. 28 %; *P* < 0.05) than subjects without *IKZF1* deletion.

**Table 1:** Subject- and disease-related variables

Variable	All subjects	*IKZF1*-deleted	*IKZF1* wild type	*P-*value
*N*	146	68 (47 %)	78 (53 %)	
Age, y				
Median (range)	27 (14–70)	34 (15–70)	22 (14–59)	0.045
Gender				
Male	72	33 (46 %)	39 (54 %)	0.859
WBC (×10E + 9/L)				
Median (range)	10 (0.5-435)	14 (2–435)	9 (0.5-168)	0.008
Hemoglobin (g/L)				
Median (range)	90 (23–157)	92 (35–148)	89 (23–157)	0.300
Platelets (×10E + 9/L)				
Median (range)	57 (0.2–313)	58 (0.2–311)	52 (2–313)	0.600
Bone marrow blasts, %				
Median (range)	87 (28–99)	86 (28–98)	88 (28–99)	0.300
*BCR-ABL1*	52	39 (75 %)^a^	13 (25 %)^b^	0.000
Cytogenetics				
Normal	47	19 (40 %)	28 (60 %)	0.305
Abnormal	81	38 (47 %)	43 (53 %)	
t(9;22)	43	31 (72 %)	12 (28 %)	0.000
t(X;2)(p22;p21)	1	1	0	
del(9)(p13)	1	1	0	
inv(9)	2	1	1	
47,+X	1	1	0	
Complex	33	3	30	
Unknown	18	11 (61 %)	7 (39 %)	

^a^8 subjects with *BCR-ABL1* had no t(9;22) including 5 with insufficient metaphases, 2 with an abnormal karyotype but not t(9;22) and 1 with a normal karyotype

^b^1 subject with *BCR-ABL1* had a normal karyotype

### Outcomes and *IKZF1* deletion

**Fig. 1:**
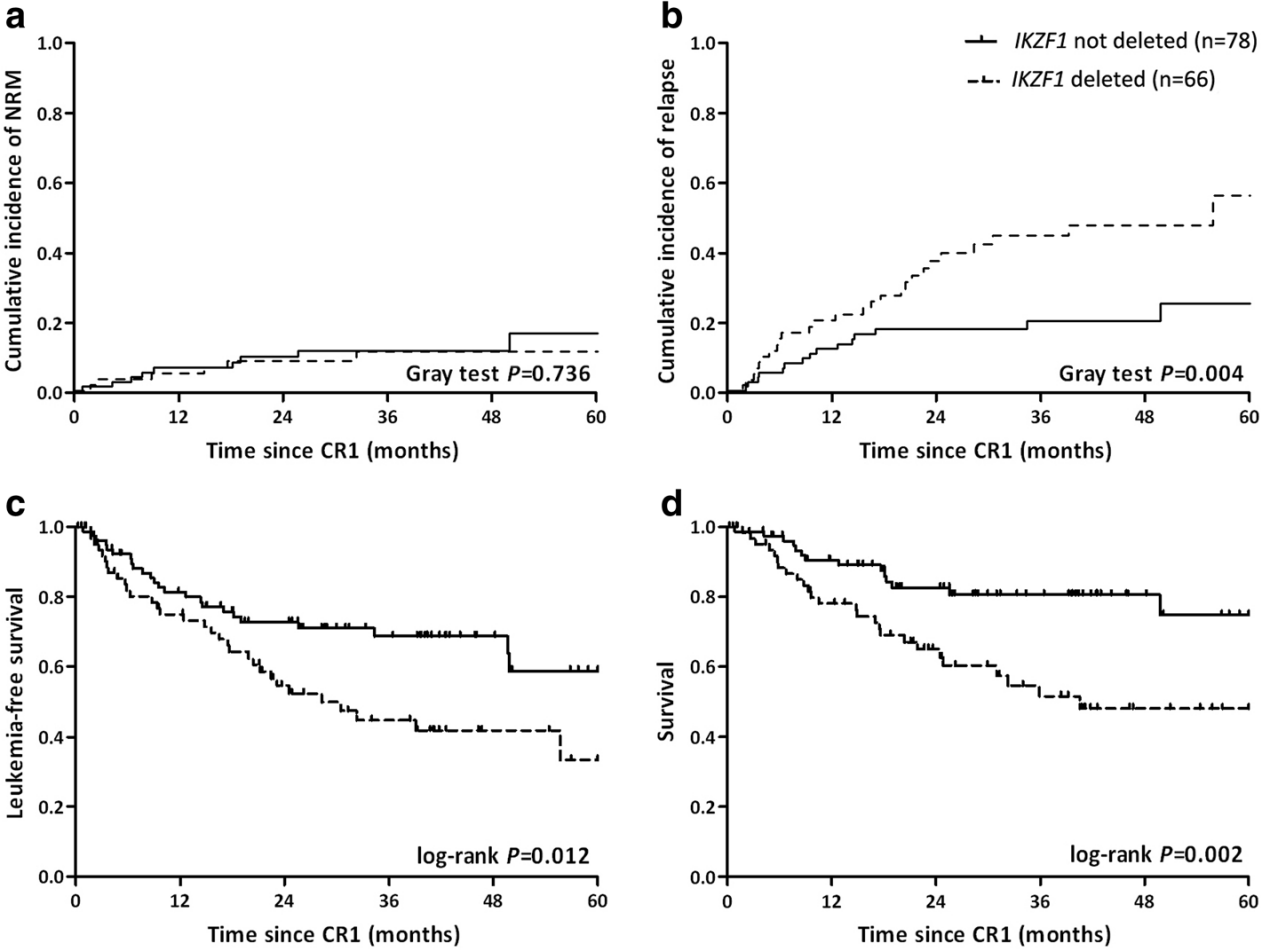
**a** Cumulative incidence of non-relapse mortality (NRM); **b** cumulative incidence of relapse (CIR); **c** leukemia-free survival (LFS); and **d** survival of subjects with and without *IKZF1* deletion since first complete remission

Median follow-up was 22 months (range, 2–91 months). 146 (90 %) subjects achieved complete remission. 2 subjects in the *IKZF1* cohort relapsing during consolidation chemotherapy and before they could be assigned to further chemotherapy-only or an allotransplant were excluded from further analyses. 5-year NRM in the 144 subjects was 14 % (95 % confidence interval [CI], 6–22 %), 5-year CIR was 38 % (27–49 %), 5-year LFS, 48 % (36–59 %) and 5-year survival, 63 % (51–72 %). In subjects with *IKZF1* deletion 5-year NRM was 11 % (2–20 %) vs. 16 % (4–28 %; *P* = 0.736, Fig. 1a) for subjects without *IKZF1* deletion. In subjects with *IKZF1* deletion 5-year CIR was 55 % (35–76 %) vs. 25 % (12–38 %; *P* = 0.004; Fig. 1b) for subjects without *IKZF1* deletion. In subjects with *IKZF1* deletion 5-year LFS was 33 % (16–52 %) vs. 59 % (42–73 %; *P* = 0.012; Fig. 1c) in subjects without *IKZF1* deletion. In subjects with *IKZF1* deletion 5-year survival was 48 % (33–62 %) vs. 75 % (57–86 %; *P* = 0.002; Fig. 1d) in subjects without *IKZF1* deletion. There were 26 deaths in *IKZF1* deletion cohort, 20 from leukemia and 6 from other causes. There were 15 deaths in the no *IKZF1* deletion cohort, 5 from leukemia and 10 from other causes. Prognoses were similar in subjects with a dominant negative *IKZF1* isoform compared to haplotype-insufficient subjects: 4-year NRM 14 % (1–27 %) vs. 8 % (0–19 %; *P* = 0.32); 4-year CIR 50 % (29–70 %) vs. 44 % (23–64 %; *P* = 0.54); 4-year LFS 36 % (18–55 %) vs. 49 % (28–67 %; *P* = 0.20) and 4-year survival 45 % (23–65 %) vs. 51 % (30–69 %; *P* = 0.53).

Because *IKZF1* deletion and *BCR-ABL1* were confounded (*r* = 0.407; *P* < 0.001), we analyzed the impact of *IKZF1* deletion in subjects with and without *BCR-ABL1*. Amongst the 94 subjects without *BCR-ABL1* the 65 subjects with *IKZF1* deletion had a significantly higher 4-year CIR than the 29 subjects without *IKZF1* deletion (51 % [30–72 %] vs. 17 % [7–28 %]; *P* = 0.002). These subjects also had worse LFS (33 % [15–52 %] vs. 72 % [58–82 %]; *P* = 0.001) and worse survival (46 % [24–66 %] vs.84 % [71–91 %]; *P* = 0.000). There was no significant difference in their NRM (17 % [1–32 %] vs. 11 % [2–19 %]; *P* = 0.525). In contrast, there were no significant differences in NRM, CIR, LFS or survival in subjects with *BCR-ABL1* with and without *IKZF1* deletion.

### Interaction between *IKZF1* deletion and post-remission therapy

**Fig. 2:**
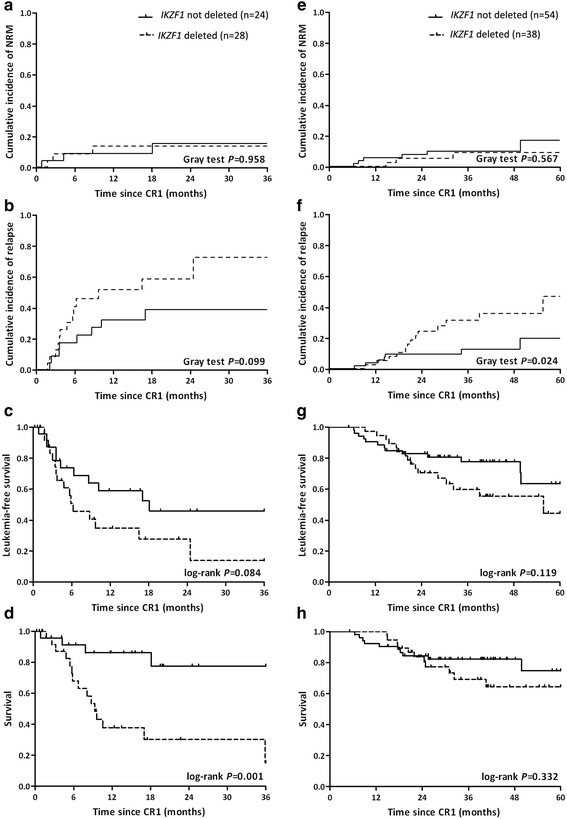
NRM, CIR, LFS and survival of subjects with and without *IKZF1* deletion receiving chemotherapy-only (**a**-**d**) or an allotransplant (**e**-**h**) since 1^st^ complete remission. Abbreviations: NRM, non-relapse mortality; CIR, cumulative incidence of relapse; LFS, leukemia-free survival; CR1, 1^st^ complete remission

Fifty-two subjects achieving remission received chemotherapy-only. 3-year NRM was 15 % (3–26 %), 3-year CIR, 53 % (36–70 %), 3-year LFS, 32 % (17–49 %) and 3-year survival, 44 % (23–64 %). Subjects with *IKZF1* deletion (*N* = 28) had similar 3-year NRM (14 % [0–29 %] vs. 15 % [0–33 %]; *P* = 0.958), 3-year CIR (73 % [41–100 %] vs. 39 % [16–61 %]; *P* = 0.099), worse 3-year LFS (14 % [1–41 %] vs. 46 % [23–66 %]; *P* = 0.084) and worse 3-year survival (15 % [1–44 %] vs.78 % [48–92 %]; *P* = 0.001; Fig. 2a-d) compared with subjects without *IKZF1* deletion.

Ninety-two (64 %) subjects achieving remission received an allotransplant. 5-year NRM was 14 % (4–24 %), 5-year CIR, 31 % (17–45 %), 5-year LFS, 56 % (40–70 %) and 5-year survival, 71 % (56–81 %). Subjects with *IKZF1* deletion (*N* = 38) had a significantly higher 5 year CIR (47 % [21–73 %] vs. 20 % [3–36 %]; *P* = 0.024) but similar 5 years NRM (9 % [0–19 %] vs. 17 % [1–33 %]; *P* = 0.567), 5-year LFS (44 % [21–66 %] vs. 64 % [40–80 %]; *P* = 0.119) and 5-year survival (64 % [44–79 %] vs.75 % [53–88 %]; *P* = 0.332; Fig. 2e-h).

### Multivariate analyses

*IKZF1* deletion was independently associated with an increased relapse risk (*RR* = 2.7 [1.4–5.2]; *P* = 0.002), a greater risk of treatment-failure (*RR* = 2.1 [1.2–3.6]; *P* = 0.007) and a greater risk of death (*RR* = 2.8 [1.5–5.5]; *P* = 0.002). An adverse impact of *IKZF1* on survival was observed in subjects receiving chemotherapy-only (*RR* = 6.5 [2.1–19.7]; *P* = 0.001) but not those receiving an allotransplant (*RR* = 1.5 [0.7–3.6]; *P* = 0.336). Other variables associated with relapse were chemotherapy-only (*RR* = 6.0 [3.1–11.6]; *P* < 0.0001) and male gender (*RR* = 2.0 [1.1–3.9]; *P* = 0.030). Chemotherapy-only was also associated with greater risk of treatment-failure (*RR* = 4.4 [2.6–7.6]; *P* < 0.0001) and death (*RR* = 4.1 [2.1–7.8]; *P* < 0.0001). Age ≥35 years was the only variable associated with NRM (*RR* = 3.0 [1.1–8.7]; *P* = 0.039). Results of multivariate analyses are shown in Table 2.

**Table 2:** Multivariate analyses of NRM, relapse, treatment-failure and death

Outcome	*N*	RR (95 % CI)	*P*
NRM			
*IKZF1* deleted vs. not deleted	66/78	0.8 (0.3–2.2)	0.630
Age: ≥ vs. < 35 y	55/89	3.0 (1.1–8.7)	0.040
Relapse			
*IKZF1* deleted vs. not deleted	66/78	2.7 (1.4–5.2)	0.002
Chemotherapy vs. transplant	52/92	6.0 (3.1–11.6)	0.000
Male vs. female	73/71	2.0 (1.1–3.9)	0.030
Treatment-failure			
*IKZF1* deleted vs. not deleted	66/78	2.1 (1.2–3.6)	0.007
Chemotherapy vs. transplant	52/92	4.4 (2.6–7.6)	0.000
Death			
*IKZF1* deleted vs. not deleted	66/78	2.8 (1.5–5.6)	0.002
Chemotherapy vs. transplant	52/92	4.1 (2.1–7.8)	0.000
Chemotherapy			
*IKZF1* deleted vs. not deleted	28/24	6.5 (2.1–19.7)	0.001
Transplant			
*IKZF1* deleted vs. not deleted	38/54	1.5 (0.7–3.6)	0.336

*Abbreviation*: *NRM* non-relapse mortality, *RR* relative risk, *CI* confidence interval

## Discussion

*IKZF1* deletion was independently-associated with higher risks of relapse, treatment-failure and death in adults with common B-cell ALL compared with similar subjects without *IKZF1* deletion. Although *IKZF1* deletion was confounded with *BCR-ABL1*, the adverse impact persisted after adjusting for *BCR-ABL1* and parallels similar findings in children with B-cell ALL [12, 15]. The adverse impact of *IKZF1* deletion operated mainly in subjects without *BCR-ABL1*. In subjects receiving an allotransplant the adverse impact of *IKZF1* deletion was less than in those receiving chemotherapy-only suggesting a benefit for using transplants in subjects with *IKZF1* deletion.

Our analysis was complex because of confounding between *IKZF1* deletion, *BCR-ABL1* and post-remission therapy (chemotherapy-only vs. an allotransplant). We used multivariate analyses to resolve this confounding. We found *IKZF1*deletion, chemotherapy-only, gender and age were significantly associated with outcomes whereas other variables including WBC, cytogenetics and *BCR-ABL1* were not. This is surprising, especially the lack of a significant association between *BCR-ABL1* with outcomes. There are several possible explanations. One is insufficient statistical power; there were relatively few subjects with adverse cytogenetics, hypo-diploidly or a complex karyotype. However, low power cannot explain the lack of a significant association between *BCR-ABL1* and outcomes. As indicated, *IKZF1* deletion and *BCR-ABL1* were confounded. Subjects with *BCR-ABL1* received different initial therapy than subjects without *BCR-ABL1* including TKIs in 90 % and an allotransplant in 58 %. If these interventions were highly-effective they could mitigate the adverse impact of *BCR-ABL1*. This notion is supported by the observation the adverse predictive impact of *IKZF1* deletion was detected in subjects without but not in those with *BCR-ABL1* and in subjects receiving chemotherapy-only but not in those receiving an allotransplant.

Our conclusions contrast with data from a study of 83 subjects with *BCR-ABL1* which reported *IKZF1* deletions were associated with more relapses and worse LFS [16]. However, there are several important differences in subjects (we studied only subjects with common B-cell ALL) and post-remission therapy (few of their subjects received an allotransplant). Our conclusion is similar to reports in children with *BCR-ABL1* in whom *IKZF1* deletion is associated with worse outcomes and with results of a recent meta-analysis indicating *IKZF1* deletion is independently-associated with worse outcomes in children and adults with ALL [27, 28].

There are several limitations to our study. One, as discussed, is confounding between *IKZF1* deletion, *BCR-ABL1* and post-remission therapy. Confounding is difficult to satisfactorily sort out in multivariate analyses but our data suggest that *IKZF1* deletion was independently-associated with higher risks of relapse, treatment-failure and death in subjects without *BCR-ABL1*. Second, subjects with *BCR-ABL1* received different induction, consolidation and post-remission therapies than those without *BCR-ABL1*. However, therapy-assignment was made without knowing *IKZF1* deletion data and we tried to account for this complexity in multivariate analyses. Another limitation is we did not include several potentially important variables (CNS leukemia, *MLL* rearrangement, *E2A-PBX1* transcripts and TKI therapy) in the multivariate analyses because of too few subjects or missing data. The sum of these potential limitations means our conclusions require confirmation in prospective, randomized studies.

## Conclusion

In adults with common B-cell ALL achieving complete remission *IKZF1* mutation was independently-associated with a higher CIR and worse survival than subjects without *IKZF1* mutation. These differences were greater in subjects receiving post-remission chemotherapy than in subjects receiving an allotransplant. We suggest a randomized trial to confirm our observation.

## Abbreviations

MLPA: multiplex ligation-dependent probe amplification
NRM: non-relapse mortality
CIR: cumulative incidence of relapse
LFS: leukemia-free survival
ALL: acute lymphoblastic leukemia
CNS: central nervous system
ROC: receiver-operator characteristic
MRD: measureable residual disease
TKI: tyrosine kinase-inhibitor
CR: complete remission
RQ-PCR: quantitative real-time polymerase chain reaction
